# Exploring the Perceived Barriers to Following a Mediterranean Style Diet in Childbearing Age: A Qualitative Study

**DOI:** 10.3390/nu10111694

**Published:** 2018-11-06

**Authors:** Harriet Kretowicz, Vanora Hundley, Fotini Tsofliou

**Affiliations:** Department of Human Science and Public Health, Centre for Midwifery, Maternal and Perinatal Health, Faculty of Health and Social Sciences, Bournemouth University, Bournemouth BH1 3LT, UK; s4933331@bournemouth.ac.uk (H.K.); vhundley@bournemouth.ac.uk (V.H.)

**Keywords:** Mediterranean diet, barriers, dietary change, childbearing age

## Abstract

A considerable amount of research has focused on interventions in pregnancy to promote health in current and future generations. This has yielded inconsistent results and focus has turned towards improving health in the preconception period. Promotion of healthy dietary patterns similar to a Mediterranean diet in the preconception years has been suggested as a dietary strategy to prevent maternal obesity and optimize offspring health. However, it is uncertain whether adoption is acceptable in women of childbearing age. This qualitative study aims to investigate the perceived barriers to following a Mediterranean diet in women of childbearing age. Semi-structured focus groups were used to generate deep insights to be used to guide the development of a future intervention. Nulliparous women aged between 20 and 47 years were recruited (*n* = 20). Six focus groups were digitally audio recorded and transcribed verbatim by the researcher. Thematic analysis was used to analyze data, which occurred in parallel with data collection to ascertain when data saturation was reached. Five core themes were identified: Mediterranean diet features, perceived benefits, existing dietary behavior and knowledge, practical factors, and information source. The present study highlights that a Mediterranean diet is acceptable to childbearing-aged women, and the insights generated will be helpful in developing an intervention to promote Mediterranean diet adoption.

## 1. Introduction

It is well established that the health of an expectant mother can significantly influence the health and wellbeing of the developing baby [1,2]. Nutrition is a major factor in optimizing health, and the impact of maternal nutrition on offspring has gained considerable attention in recent years. The ground for a focus on maternal nutrition are threefold: first, a large body of evidence from epigenetics suggests maternal nutrition can affect the body composition and metabolic health of the developing baby, at birth and in later life [3,4]; second, maternal obesity and excessive gestational weight gain are associated with a number of adverse pregnancy complications, including miscarriage [5], still birth [6], preeclampsia and hypertensive disorders [7], gestational diabetes mellitus (GDM) [8,9], emergency caesarean section [10], macrosomia [11], and preterm delivery [12] amongst others, and rates have doubled in the past two decades [13]; and finally, nutrition is a largely modifiable factor with an underestimated potential to provide significant improvement to both the short and long term health of mother and baby, and a reduction in pregnancy complications.

Several interventions have been developed with the aim of improving nutrition in pregnancy to minimize complications, improve offspring and maternal health, and to limit gestational weight gain [14]. However, while some trials have shown positive trends in eating behavior among obese pregnant women [15,16], a lack of beneficial clinical outcomes and an average reduction in gestational weight gain by a mere 0.7 kg [14] has made it difficult to draw firm conclusions about the efficacy of pregnancy interventions [17]. It is likely that intervention in pregnancy may be necessary to mitigate problems associated with poor maternal nutrition, but additional interventions are needed to optimize health and nutrition and prevent obesity prior to conception.

Promoting health and optimizing nutrition prior to pregnancy is supported by research that has found diet quality and health long before conception can also influence the health of future offspring and pregnancy outcomes [18,19,20,21]. However recent evidence suggests that women across the span of childbearing age from late adolescence up to 49 years [22] are not nutritionally prepared for pregnancy, with 9 in 10 eating fewer than five fruit and vegetable portions a day, consuming 50 g of free sugars daily on average, consuming only 17 g of fiber daily on average, and nearly 6 in 10 with folate levels below the clinical threshold [23]. In addition, it is unlikely that pregnancy is planned for years prior to conception and is often unexpected, allowing for sub-optimum nutrition status of the mother [23]. This, together with evidence suggesting that the gestation period may be too short to demonstrate significant behavior changes and the mixed results of clinical efficacy of interventions in pregnancy, suggests a need and potential benefit for interventions in the childbearing age population in general, and there is an emerging consensus that this strategy should be pursued [17]. In order to optimize the nutritional health in women prior to pregnancy, a supportive, evidence-based, and pragmatic method of nutrition and health promotion must be decided upon. Research into pre-pregnancy dietary patterns and health has consistently shown a dietary pattern which is high in vegetables, fruits, wholegrains, lean proteins, and fish, and low in red and processed meats and refined sugary foods is associated with positive health outcomes for both mother and offspring, and therefore should be adopted [19].

The Mediterranean diet (MD) is such a dietary pattern, and is characterized by high consumption of vegetables, fruits, legumes, extra virgin olive oil as the principal fat source, a moderate consumption of fish, lean protein, and dairy, and low consumption of processed foods and sweets [24]. The MD has been shown to prevent diseases associated with chronic inflammation, including coronary heart disease and stroke, type 2 diabetes, cognitive diseases, and obesity [25]. In addition, research has also demonstrated numerous benefits to the preconception and pregnancy periods, such as a reduced risk of hypertensive disorders [26], reduced risk of GDM [27], improvement in glucose tolerance [28], reduced gestational weight gain [29], reduction in depressive symptoms and risk of postnatal depression [30], and improved offspring homocysteine and lipoprotein levels [31]. 

It is therefore possible that following an MD pattern may have the potential to boost the overall health in women of childbearing age, whilst reducing the risk of complications during pregnancy and optimizing the health of mother and baby, if or when pregnancy should arise in the future. Yet, there are concerns relating to the feasibility of promoting the MD to young women in the U.K. and other non-Mediterranean populations and the acceptability to individuals currently consuming a Western diet [32,33], as there is evidence suggesting that low adherence to an MD is common in younger generations, with a decline in adherence even in Mediterranean countries [34,35]. However, prior research has shown the diet pattern to have high palatability, adherence, and transferability, even in non-Mediterranean countries [36,37,38,39]. To the best of our knowledge, there is no existing research that investigates the feasibility of promoting a Mediterranean dietary pattern to women of childbearing age. In order to utilize the Mediterranean diet as a way of promoting health and wellbeing and create the most effective, informative, and appealing intervention, it is necessary to develop a deep and thorough understanding of the possible barriers to following this dietary pattern and the ways to overcome them in this population of women.

The primary aim of the study was to explore barriers and enablers to following a Mediterranean style diet in women of childbearing age in the U.K.

## 2. Methods

A qualitative design was utilized. Semi-structured focus groups were chosen to gain a detailed insight into perceived barriers and enablers to following a Mediterranean style diet. This method was chosen because participants were able to give their own thoughts and build on the views of others, thus generating broad and in-depth discussions [40]. This method has been used in a wide range of health research, including to assess attitudes relating to lifestyle change [41], to identify health needs in specific populations [42], to investigate the feasibility and acceptability of behavioral interventions [43], and to also explore experiences of health interventions [44], as it is essential that a thorough understanding of the target population is gained [45].

The study utilized a convenience sample of university students and employed women identified through a snowballing approach [46]. Women aged 18–49 years old, who were U.K. residents, nulliparous, had low to medium MD adherence, and did not study or have an occupation directly related to nutrition were eligible to participate in the study. Participants were recruited via posters, social media, email, and in person. The research was undertaken between May and June 2017 and was approved by the Bournemouth University Research Ethics Committee.

Thirty-eight participants were initially recruited for the study. Three participants were ineligible due to parity and residency status. A further 15 participants withdrew prior to participation, leaving 20 in total, engaging in six focus groups.

Prior to focus group participation, participants were sent a document containing detailed information about the study and a link to a two-part online questionnaire which was used to confirm eligibility. Part one related to general information age, socioeconomic status, and parity, and required participants to self-report their height and weight. Participant characteristics are summarized in Table 1.

**Figure 1:**
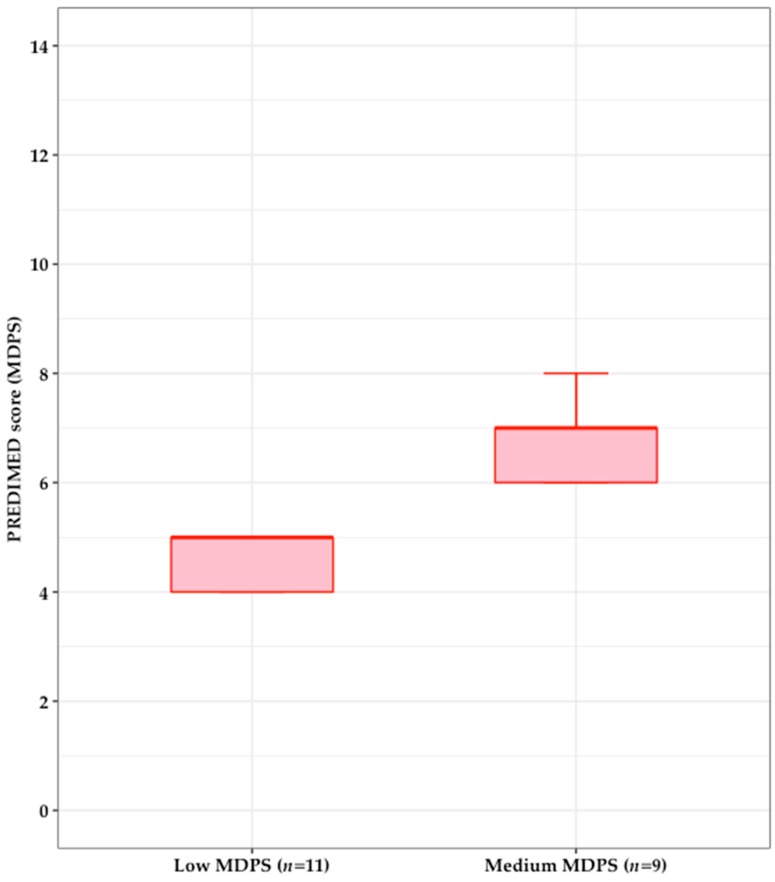
Participants’ adherence to the MD assessed using the PREDIMED score (MDPS). In the boxplot showing the distribution of PREDIMED score (MDPS), eleven participants scored low on MDPS (≤5) (median 5, range 4–5), and nine participants had scores in medium tertile (median 7, range 6–8). The dark red horizontal line is the median.

Part two assessed MD adherence using the validated 14-item PREvencion con DIetaMEDiterranea (PREDIMED) score (MDPS) [47]. The range of possible scores for MDPS is 0–14, and those who had a high adherence (≥10) would not be eligible for participation as they were not considered to be inclusive in the target population. Figure 1 shows the distribution of participants’ scores for MDPS. Overall mean MDPS was 5.6 ± 1.3; none of the twenty participants demonstrated high MD adherence, so all were included in the focus groups.

The focus groups were led by a facilitator and conducted in a private room. The focus group procedure was explained and participants were reminded of the audio recording and their right to withdraw. 

Participants were sent an MD information sheet, including an MD pyramid demonstrating the types and quantities of food and drink recommended in the diet patter, as well as information relating to the health benefits, based on current evidence. This allowed participants to familiarize themselves with the content of the dietary pattern and provided basic information in preparation for the focus groups. This information was also available for reference throughout the focus group process. Open-ended questions were used to guide discussions.

A semi-structured focus group guide was used with open-ended questions and probes allowing for the exploration of possible barriers to following an MD, with indicative topics including awareness of MD, motivations and eating behaviors, practicalities and social perception, and source preferences (Appendix A). Participants were given the opportunity to add or raise any additional points before concluding the focus groups, and on completion participants received a £5 gift voucher.

The digital audiotapes were transcribed verbatim by the facilitator (H.K.) after each focus group. Data collection and analysis co-occurred to asses when data collection could be concluded once emerging themes had reached saturation. This is a process whereby no new themes are generated from the data and there is repetition in emerging ideas. Focus group transcripts were analyzed using the process of inductive thematic analysis, as described by Braun and Clarke [48]. After the initial analysis, a second researcher (F.T.) reviewed the transcripts and independently coded for themes. Any discrepancies were resolved through discussion and additional review of the transcripts.

## 3. Results

The study identified five core themes encompassing perceived barriers and enabling factors to following a MD and include: MD features, perceived benefits, existing dietary behavior and knowledge, practical factors, and information source. Themes are illustrated with representative verbatim quotations from participants, represented numerically to protect anonymity, followed by participant age (e.g., (P. (*n*)/age (years))).

### 3.1. Mediterranean Diet Features

Several participants reported barriers and enablers relating to features of a MD. Two main subthemes were identified as the components of the MD and its perceived qualities. Participants indicated that a reduction in meat and an increase in plant-based foods would be difficult (“*I eat a lot of red meat, that’s what I would find hard, making it more plant-based*” (P. 03/21)), and suggested the components of the diet were unappealing (“*it just looks quite bland…the lack of meat and like lentils and stuff, it’s a bit off-putting, a bit lacklustre*” (P. 14/23)). Participants also believed that the low inclusion of sweet treats would also pose a barrier: “*not eating processed or refined stuff, or stuff high in sugar, I think I would really, really struggle with that*” (P. 18/21).

The perceived qualities of an MD were identified and considered to be enabling by many participants and included the non-restrictive nature of the diet: “*It’s quite diverse, and there’s quite a lot of different things you can eat, and normally you’re quite restricted but it seems like most food groups are involved in the diet*” (P. 13/20). The description of the MD as a “diet” was seen to imply a negative quality to the diet, due to participants’ existing experience and understanding of the term. The term “diet” elicited ideas of restriction, and many participants found its use off-putting in this context (“*when you say diet, you think of restrictions*” (P. 20/23)), and not conducive to the ethos of the MD: “*The word diet makes you automatically think losing weight, but I think whether it’s a change in wording or you call it the Mediterranean lifestyle, because it is about physical activity, socializing, and rest and everything, and take the focus away from food…it’s the diet word I think that puts people off, it’s a lot of restriction isn’t it*” (P. 14/23).

### 3.2. Perceived Benefits

Participants verbalized several motivations to following a healthy diet and subthemes of appearance, disease prevention, and psychological benefits were identified. Appearance was a commonly identified motivator, particularly in participants under 30 years of age, and it was suggested that benefits of a healthy diet for physical appearance were prioritized over health: “*I think of image and fitness, I probably should be but I don’t feel like things like heart disease and stroke, I don’t think about that at the moment, I think more about fitness and what I am going to look like in a bikini*” (P. 17/25).

It was therefore suggested that to enable adoption of an MD in this group that appearance benefits should be promoted rather than health benefits: “*It might even have to go down the route of ‘you will have nice hair if you have this meal’ or ‘you will have good skin’ and again focusing more on the looks, rather than what it should be about with the whole, actual health and heart disease and stroke and things like that*” (P. 20/23).

In addition to appearance being a primary motivating benefit to following a healthy diet, a significant motivating factor was the identification of disease prevention (“*I want to prevent things before they’re at risk, you think I don’t want diabetes so I’m going to change this, what I’m eating, I don’t want to have heart disease when I’m older so I am going to think about this when I am eating*” (P. 17/25)), and psychological health (“*For me, when I am eating properly I’m less stressed…it’s more my mental health that I do it for*” (P. 20/23)).

Whilst promoting an MD for the benefits of disease prevention was considered enabling, promoting it in this group of women as a method of optimizing the health of their future offspring was considered off-putting, with participants suggesting that such a focus places too much responsibility on women: “*I’m going to take quite a strong feminist view here, this is promoting it completely at women…to put all the emphasis on the mother eating healthily and the mother eating this diet for the sake of a baby seems an uncomfortable way of framing it for me*” (P. 06/42).

It also makes assumptions about the female role: “You feel kind of like a baby making machine, if they’re like ‘you need to do this because it will be good for your pregnancy’ and just not highlight other things and be like actually this is really good for your health in the long term” (P. 05/22).

### 3.3. Existing Dietary Behavior and Knowledge

A common theme of existing dietary behavior and knowledge arose. Within this, dietary habits, nutritional awareness, and familial influences were identified.

Dietary habits were either a barrier or enabler depending on individual circumstance and habit: “I know in other circumstances where people are brought up where take-aways are a regular occurrence, and I think to change from that to this sort of diet would be very difficult” (P. 12/21); “I think people who eat quite a normal diet, or healthy diet anyway, would be more likely to make changes” (P. 09/42).

Existing dietary information was perceived as a significant barrier to following an MD, with participants expressing confusion with available dietary information and a difficulty in knowing which advice to follow: “*It sounds like you get a lot of benefits from the same diets and it gets really quite confusing, whenever I look up what’s good for you and what’s not there are cross cuts all over the place, so I never actually know what sort of diet is good for you*” (P. 12/21).

In addition, conflicting dietary health messages were considered off-putting, and it was indicated that this was a barrier to following any forms of dietary advice in general: “*There are so many different things out there, so many different diets that’s a benefit and there’s another bit of research that goes against it, and for everything they say is good, there is something else saying it’s bad, so I don’t really tend to pay too much attention*” (P. 14/23).

An absence of nutrition education was indicated as a barrier to following a MD, with the belief of a lack of taught cooking in childhood was a considerable barrier for following a healthy diet as an adult: “*People don’t have home economics or cooking at school now do they? That’s quite frightening isn’t it, so then you learn from your mother and if your mother doesn’t cook or your grandmother never cooks, you’re never going to cook*” (P. 04/47).

Specific to an MD, re-education around dietary oils was also highlighted, as current perceptions of dietary fats were suggested as a barrier: “*I think more knowledge around olive oil, because people look at oil and think, ‘Oh God, that’s going to make me fat’ so more education about why it’s good and what makes it good*” (P. 04/47). 

The impact of the influence of family members was verbalized as a possible barrier to following an MD. Some participants indicated that non-compliance from a partner would prevent personal adherence to the diet: “*I would love to cook with them [lentils and pulses] actually, I really would, but I would need to work on my other half, because he thinks a meal without meat is not a meal*” (P. 04/47); “*I think it’s important to have the same kind of behavior towards food, because if you’re around someone that has a negative relationship with food, you would be more likely to*” (P. 20/23).

Related to this, the upbringing and family values and practices around food was highlighted to be an enabler to following a Mediterranean style diet in this group of women: “*I think also because I have grown up with a healthy diet I enjoy that kind of stuff, I naturally think to put vegetables with my dinner, it’s just how I’ve been brought up*” (P. 19/21).

### 3.4. Practical Factors

Practical factors relating to following a MD were highlighted as possible barriers. Within this, cooking skills, time and convenience, seasonal influence, and affordability were identified.

Participants indicated that cooking skills would be necessary to follow an MD: “*This diet is all about non-processed, it’s all about the cooking*” (P. 06/42). This was not necessarily considered a problem in this group of women, but was suggested as a barrier for others: “*If I didn’t cook and looked at this I would think I would just have to eat salad all the time*” (P. 04/47). However, it was also discussed that the content did not appear to in fact require more culinary ability that any other food preparation: “*I don’t think it would be that different food prep from any other diet*” (P. 14/23).

Time and convenience were significant barriers for most participants, with preparation and cooking of fresh food perceived as time consuming and effortful, and therefore was not always possible due to busy lifestyles: “*I will finish work and go to the gym, so when I get home I just want to eat something that is quick*” (P. 07/33). Participants explained that if healthy food required too much time to prepare, then sometimes convenience food would be chosen instead: “*I know that if I was following a diet where every evening you had half an hour or an hour preparation, then some evenings I know I would just come home and order pizza instead*” (P. 13/20).

Seasonal influence and climate differences were suggested to have an impact on the ability to follow an MD, and participants believed that a Mediterranean climate facilitated following a MD (“*This has a big chunk of regular exercise, physical activity, socializing and rest…the weather has a big effect on that, you can socialize more outside, you can do more things outside all year around*” (P. 01/24)) and unpredictable climate was implicated in food choice (“*You might not want to eat like that in the winter, you would want a lot more warm recipes*” (P. 13/20)). Coupled with this, the seasonal availability of produce was also considered problematic: “*When it comes to fresh diets, it’s really dependent on the seasons, and it will change in the winter, it might be really hard and really expensive to get things then*” (P. 13/20).

Affordability of an MD was found to be both a barrier and enabler (“*Fruits and veg and quality meat is actually really quite expensive*” (P. 12/21)); however, the reduction in meat and increase in plant-based foods was conversely considered positively by some (“*I think fruit and vegetables can be expensive, but I think in this diet you are cutting out red meat which is also really expensive, like steak and stuff, that’s expensive, so on the whole it would balance out*” (P. 03/21)) but it was suggested that the variety in the content of an MD made it possible to tailor adoption to individual affordability: “*I don’t think it has to be expensive, eating healthy, as people think, because frozen vegetables have just as much nutritional value as fresh I think, and things like lentils and beans and pulses are very cheap*” (P. 09/42). 

### 3.5. Information Source

Participants discussed their preferences for sources of diet and health information and source type and resource features were identified. Information was often sought from multiple sources, including the internet, traditional media such as magazines, social media, word of mouth, and books, however, the use of the internet was largely the main identified source of information, either as a primary source (“*I will always go online and find everything*” (P. 10/21)), or to seek validation of information gained elsewhere (“*I go by quite a lot of word of mouth, or I read a newspaper or magazine, then I will go on the internet and have a look*” (P. 13/20)). The internet was also considered an enabler due to the immediacy of access and the amount of information available: “*I think it is probably easier now than it ever has been before because it’s so easy to find stuff online, from recipes to ‘other ways to get more olive oil into your diet’ or whatever, there’s never been a better time to do this*” (P. 09/42). Conversely however, participants also expressed distrust about existing information and preferred reputable and evidence-based sources: “*it would have to be available from a reputable source…somewhere that is not ‘The Sun’ or ‘Mail Online’*” (P. 15/21). 

Certain features were considered particularly appealing, and participants expressed a preference for blogs due to their interactivity and relatability (“*I think a blog is good…you feel like you’re reading something you can relate to*” (P. 02/24)), as well as the inclusion of recipe ideas: “*things like recipe ideas or knowing how easy it is, or how easy it is to get ingredients would be helpful*” (P. 07/33).

## 4. Discussion

Although the feasibility and acceptability of the MD has been explored in older people and those in middle age [37,38,49,50], to date there has been no research that has investigated the feasibility of promoting an MD to women of childbearing age, with only evidence from cross-sectional studies in adolescent girls and women in early adulthood suggesting poor adherence to diet patterns similar to the MD [51,52]. Promoting a healthy diet in women of childbearing age can have a significant impact on both maternal and child health; however, the present study demonstrates that whilst an MD may be generally well received, adherence would require several obstacles to be overcome. Our findings suggest that in order to achieve behavioral change there would need to be reframing of ideas about diet, removal of barriers to change, and motivational buy-in. 

One of the key points to address is how to reframe the diet in terms of language and benefits to make it more appealing. Participants’ understanding and experience of the term “diet” came with negative connotations, including restriction and dissatisfaction. The description of an MD as a “diet” is therefore potentially off-putting, especially to those who have previously undertaken restrictive dietary practices. Linked with this notion is the perception that eating Mediterranean style foods is non-restrictive, with the inclusion of all food groups described as a significant enabling factor. Previous research on the use of language and terminology in health promotion messages has shown that language choice when promoting healthy eating is of critical importance [53], and therefore promotion of a MD as a “lifestyle” which emphasizes the lack of strict restriction is likely to be more appealing to this population of women.

Although a MD was considered non-restrictive where the reduction of red meat and increase in legumes was considered by participants to be particularly challenging, which has been previously reported [33,39,49,54]. Indeed, whilst implementing MD content in non-Mediterranean countries might prove challenging, there are simple practical approaches that can be offered to individuals who wish to adopt an MD in favor of a Western diet [32,33]. Participants suggested that education around cooking more plant-based meals and the provision of recipes would help address the limitations of content changes, such as increasing the consumption of legumes while substituting red or processed meat. Several practical barriers were highlighted in the study including time, knowledge, and finances. As found in other research on MD adoption and healthy eating [38,55], a lack of time to prepare food and the perceived cost implications of purchasing healthy food were highlighted as barriers [38,55,56,57]. To overcome these factors, it was suggested that a range of recipes with varying time requirements be provided, alongside cost reducing methods in order to change the perception that a healthy diet is expensive. Examples included providing information on cost per serving, leftover ideas to reduce wastage, and options for using frozen, tinned, and dried fruit, vegetables, and legumes, which are typically inexpensive. It may also be beneficial to highlight that the cost of consuming a healthy diet with increased fruit and vegetables may be offset by a reduction in meat products, as has also been shown in previous research [58].

Climate was highlighted as a possible barrier to healthy food choice, with participants specifying the belief that eating a healthy diet was easier in warmer climates and seasons, with “comforting” recipes being preferred in colder weather. This is consistent with previous research on climate and seasonal variation in dietary intake, which has shown variations in fruit and vegetable consumption across seasons [59,60,61] although research in this area is limited and there are conflicting results as to whether variations of significance exist [62]. Climate was also indicated in part as a financial barrier, with participants indicating that fresh produce would only be seasonally available and therefore be expensive to purchase imported goods. It was suggested that this could be overcome relatively simply by providing recipes containing seasonal produce. However, a larger scale intervention with governmental support may be necessary to increase consumption of fruit and vegetables in the population in general [33]. Some countries have successfully socially engineered fruit and vegetable availability, including the North Karelia project in Finland where the Ministry of Commerce and the Ministry of Agriculture promoted domestic berry and vegetable products and encouraged farmers to switch to growing these [63]. Such population-based interventions are expensive and complex to implement, but could be necessary to overcome this barrier [33].

Aside from barriers of practicalities, a lack of external support from partners and family members was considered a barrier and the sharing of behaviors and attitudes towards diet was considered key in the facilitation of healthy lifestyle. The perceived need for partner or familial support is in accordance with other dietary intervention research that has found social support to be an important factor in successful dietary adherence [64,65,66], and therefore targeting promotion and both men and women may encourage adherence. 

Whilst the provision of recipes and dietary information may be helpful in overcoming some of the practical barriers highlighted, the provision of information alone is often insufficient to change behavior [53] Motivation is identified as a key component in health behavior change in theoretical models [67,68,69,70], and the perceived benefits of a healthy diet were motivations that emerged as a key theme. Appearance benefits were found to be a significant motivator, which supports previous research that suggests weight management and appearance benefits should be prioritized when promoting a healthy diet to young women [71]. It is possible that promoting an MD for appearance benefits may encourage adherence as well as conveying health benefits to this group of women.

Enabling factors of following an MD mostly centered around disease prevention, as has previously been shown in studies investigating MD adoption in non-Mediterranean countries [32]. Emphasis on the protective effects of the MD against non-communicable diseases (NCD) and for mental health benefits [72,73] would likely be beneficial in its promotion. Interestingly, although the health benefits of an MD were enabling factors, the reported benefits in preconception and pregnancy [26,27,28,29,30,31,74,75,76] were considered less important. Most participants indicated that the possibility of pregnancy was too far in the future to contemplate following a dietary pattern for benefits, and diet would not even be a concern in some participants unless there was difficulty in conception. Previous literature has shown that the proximity of goals can be influential in behavior change with proximal goals seen as more influential on behavior change than distant outcomes [77,78,79]. The present findings could therefore suggest that pregnancy-related benefits could be perceived as a short-term health goal, only relevant for those who are planning pregnancy. However, it is interesting to note that participants were able to conceptualize and accept that following a healthy eating pattern in the present would likely convey both short-term health benefits and NCD prevention in later life.

Whilst promotion of the diet for pregnancy benefits were perceived as irrelevant by some, they were also perceived negatively by other women, with the expression of the belief that promoting a diet at young women for pregnancy and offspring benefits places an emphasis on the reproductive role of women. This is contrary to previous research, which has found a reduced risk of health issues in pregnancy to be an enabling factor to healthy eating in young people [80]. Encouragement of preconception healthy is a priority, but it is important that pregnancy benefits are not over-emphasized to generate the perception that the MD is only applicable to those planning pregnancy.

In terms of information source of diet and health information, a range of sources were highlighted and most participants expressed a preference for use of the internet. Recipes, blogs and interactivity, photographs, and simple and clear evidence-based information were preferred, similar to features previously identified in other studies investigating acceptability of a MD [39]. The development of a future intervention promoting the MD should include these elements to assist in overcoming some of the identified barriers.

## 5. Strengths and Limitations

A thorough search of existing literature yielded no prior research investigating the perceived barriers to following an MD in women of childbearing age in the U.K., making this study the first of its kind. The qualitative methodology used enabled a deep and thorough understanding, which is recommended to aid the development of effective interventions [81] and will be used in the development of an MD-based intervention for the purposes of the promotion of optimum health in this group of women. Both university students and employed women were recruited for the study to promote heterogeneity amongst participants and to invoke a wide range of insights, and a broad age range was recruited to ensure adequate representation of the childbearing period. Inclusion of participants with only low to moderate adherence to the MD is also a significant strength to the study, as these women are the target population for a future intervention.

The limitations of the study relate to the relatively small sample size. There has been considerable discussion about the ideal size of a sample for qualitative research and, while differing views exist, a sample of 20 is usually considered adequate [82]. In addition, saturation of data was achieved in this group. The sample of women recruited for this study were well-educated, with all women educated to age 18 at a minimum and those who were not university students were employed in professional roles. This is, therefore, not representative of the wider population, and it is possible that individuals with other educational or socioeconomic backgrounds may have generated different data. In addition, even though adherence to an MD was low to moderate in all participants, all women believed that they currently followed a healthy lifestyle in general, suggesting a level of selection bias with an existing level of interest and motivation in healthy living. It is therefore possible that individuals with poor dietary habits will have generated different data to the participants in this study, and further investigation of barriers to following an MD in a broader sample of women would be warranted. Future research with different populations would be useful to identify whether barriers and facilitators differ in lower socioeconomic groups and other cultural contexts.

## 6. Conclusions

The nutrition of women of childbearing age has received considerable attention due to its modifiable nature. It has been suggested that the promotion of behavior change to follow dietary patterns similar to an MD is important because of the wide-ranging health benefits. It is necessary, however, to understand the possible barriers to following such a diet in women of childbearing age. This study identified five core themes including barriers and enablers, which should be addressed in the development of an intervention to effectively promote an encourage adherence to an MD. Based on these themes, five key points should be considered in the development of an intervention: (1) a reconsideration of language used to describe an MD and a focus on the qualities of the MD, such as its non-restrictive nature and inclusion of all food groups would likely be encouraging; (2) chronic disease prevention and long-term health benefits should be emphasized, along with the principle that a healthy diet will likely convey appearance related benefits should be highlighted to encourage adherence in this group; (3) the emphasis on benefits in pregnancy should be reserved specifically for those individuals planning pregnancy, as overemphasis could be off-putting; (4) emphasis should be placed on altering the perception that an MD is expensive and time consuming; and (5) the development of an intervention should have a strong and clear evidence base to support recommendations and elements of interactivity.

The insights gained from this formative research will be useful to assist in the development of a novel MD intervention for trial in a broad sample of women of childbearing age for the promotion of health prior to pregnancy.

## Figures and Tables

**Table 1:** Participants’ Characteristics.

Demographic Data	Mean (SD)
Age (years), *n* = 20	26.8 (9.4)
BMI (kg/m^2^)	23 (4)
	*n*
Employment Status	
University Student	13
Employed Non-Student	7
	*n*
Ethnicity	
White British	18
White Other	2
Median Mediterranean Diet Score (0–14)	5

## Appendix A. Focus Group Guide Which Was Used to Inform the Focus Groups

**Guide Questions & Probes**
Tell me what you previously knew about the Mediterranean diet.
How is it different to other diets you are aware of?
How do you think the known benefits would affect you and people like you?
If you were thinking about getting pregnant, when do you think you would start thinking about diet?
How do you think the known health benefits in pregnancy would affect you and people like you?
How would it affect you if this information was more widely available?
What are your thoughts about framing this diet specifically at young women?
What are your current motivations for following a healthy lifestyle?
How do you feel about the food in the Mediterranean diet in comparison to your usual diet?
Tell me what you feel about following this diet in your current lifestyle.
What are your thoughts about preparing the food in this diet?
Tell me what you think about the cost of this diet.
Tell me about how you normally find and access information on diet and lifestyle.
Tell me about features of resources that you find particularly helpful or unhelpful.
